# Health effects of milder winters: a review of evidence from the United Kingdom

**DOI:** 10.1186/s12940-017-0323-4

**Published:** 2017-12-05

**Authors:** Shakoor Hajat

**Affiliations:** Department of Social & Environmental Health Research, London School of Hygiene & Tropical Medicine, 15-17 Tavistock Place, London, WC1H 9SH UK

**Keywords:** Cold, Winter, Temperature, Mortality, Morbidity, Climate change, United Kingdom

## Abstract

Cold-related mortality and morbidity remains an important public health problem in the UK and elsewhere. Health burdens have often reported to be higher in the UK compared to other countries with colder climates, however such assessments are usually based on comparison of excess winter mortality indices, which are subject to biases. Daily time-series regression or case-crossover studies provide the best evidence of the acute effects of cold exposure. Such studies report a 6% increase in all-cause deaths in England & Wales for every 1 °C fall in daily mean temperature within the top 5% of the coldest days. In major Scottish cities, a 1 °C reduction in mean temperature below 11 °C was associated with an increase in mortality of 2.9%, 3.4%, 4.8% and 1.7% from all-causes, cardiovascular, respiratory, and non-cardio-respiratory causes respectively. In Northern Ireland, a 1 °C fall during winter months led to reductions of 4.5%, 3.9% and 11.2% for all-cause, cardiovascular and respiratory deaths respectively among adults. Raised risks are also observed with morbidity outcomes. Hip fractures among the elderly are only weakly associated with snow and ice conditions in the UK, with the majority of cases occurring indoors. A person’s susceptibility to cold weather is affected by both individual- and contextual-level risk factors. Variations in the distributions of health, demographic, socio-economic and built-environment characteristics are likely to explain most differences in cold risk observed between UK regions. Although cold-related health impacts reduced throughout much of the previous century in UK populations, there is little evidence on the contribution that milder winters due to climate change may have made to reductions in more recent decades. Intervention measures designed to minimise cold exposure and reduce fuel poverty will likely play a key role in determining current and future health burdens associated with cold weather.

**Electronic supplementary material:**

The online version of this article (10.1186/s12940-017-0323-4) contains supplementary material, which is available to authorized users.

## Background

Cold-related mortality and morbidity remains an important public health problem in the UK and many other parts of the world. Wintertime health burdens are often reported to be higher in the UK compared to other countries with colder climates which, among other reasons, suggests that impacts are preventable with appropriate adaptation measures. Very few of the health impacts are as a direct result of hypothermia, but rather due to cardiovascular and respiratory problems. Climate change will likely alter current patterns of cold-related mortality and morbidity. Other factors will also determine future health burdens, including future increases in fuel prices leading to people experiencing greater difficulties in adequately heating their homes during winter months.

This narrative review summarises the epidemiological evidence on the health effects of wintertime weather. The review is restricted to the literature on UK populations but the evidence is likely to be generalisable to other high-income settings. It identifies individual- and contextual-level risk factors that heighten vulnerability to cold and reviews the evidence for past changes in cold risk due to recent climate changes, and well as likely future burdens. The paper discusses the potential for current and future cold impacts to be reduced by adaptation measures and concludes by identifying gaps in current knowledge.

## Review

### Evidence of current sensitivity to weather factors

#### Epidemiologic studies of cold-related health

Epidemiology studies generally quantify one of two different measures of cold-related health. One relates to assessment of the seasonal distribution of health events, whereby an excess wintertime effect is obtained by measuring the number of health events occurring in the winter months compared to at other times of the year. The most commonly used metric is the Excess Winter Mortality (EWM) Index, which considers the average daily number of all-cause deaths occurring during December-March (for northern hemisphere countries) compared to the average daily number occurring in the preceding August-November and the following April-July [1]. This excess is observed to be high in the UK, Ireland and Portugal compared to many other parts of Europe, including the colder Scandinavian countries [2, 3]. However, international comparisons can be difficult to interpret due to differences in the seasonal distribution of deaths between countries – for example, in England the cold period is largely concentrated in the months of December to March, which are the months used in the numerator of the EWM index, whereas in colder countries the cold period lasts longer. The index is also difficult to interpret in other ways – cold-related deaths also occur outside of the Dec-March period, which biases the EWM since they contribute to the denominator of the calculation. Seasonal factors other than weather may also contribute to the winter excess (e.g. influenza activity) and the index can also be influenced by unusual mortality patterns at other times of the year used in the comparison months, such as deaths during a heat-wave. The main advantages and disadvantages of using excess winter mortality indices to determine wintertime health burdens are listed in Table 1 [4].

**Table 1:** Advantages and disadvantages of using an excess winter mortality index to determine wintertime health burdens

Advantages:	Disadvantages:
• It is easy and quick to calculate. If daily or even seasonal mortality counts are available, the formula can be applied without advanced statistical methods. • It is readily understood by the lay public and policymakers. • It can measure the full excess at wintertime, without missing many deaths due to delayed effects.	• It is crude. Seasonal changes in health do not occur according to fixed calendar dates. • Not all deaths due to cold are during the winter, and not all of the winter excess is due to cold. • International comparisons are complicated by potential differences in the distribution of wintertime deaths and the length of the winter period. • The ratio may be sensitive to the periods used in the denominator, e.g. if summer months are included the occurrence of a heat-wave may artificially reduce the index. • It doesn’t allow for identification of which weather factors are important for health or for the specific conditions, e.g. are cold-spells occurring early in the winter worse than later ones?

For the reasons listed, the EWM index is not an appropriate indicator of health burdens associated with wintertime weather. Indeed, there is very little correlation between recent Office for National Statistics (ONS) reports of annual excess winter deaths for England and Wales and the severity of wintertime weather experienced. Analysis of EWM to draw conclusions about cold-related health impacts can therefore lead to spurious findings [5].

By contrast, the second type of epidemiologic evidence measures the explicit effects of weather on health whilst controlling for underlying seasonal patterns. These studies are usually based on time-series regression analysis, which requires data consisting of sequences of health events and exposures measured at regular intervals of time, for example the daily number of deaths in relation to daily fluctuations in temperature in a particular location over a period of a few years [6]. An alternative study design is the case-crossover approach [7]. Because these type of studies provide the best evidence of acute effects of cold exposure, they are the focus for the literature reviewed in this paper.

##### Mortality & morbidity outcomes

Time-series regression and case-crossover studies show that in many settings there is a U- or a V-shaped relationship between year-round ambient temperature and health, with the number of adverse impacts occurring in a gradual, often linear, fashion in relation to a fall in temperature during winter months. Cold effects are therefore often presented as an increased risk in mortality/morbidity per unit decrease in the temperature measure. The strength of the cold effect may vary across cities and countries depending on differing climatic, demographic and socio-economic profiles. In UK specific studies, a 6% increase in all-cause deaths was observed in England & Wales for every 1 °C fall in daily mean temperature within the top 5% of the coldest days of the year, and deaths from respiratory diseases were the most sensitive among these [8]. In major Scottish cities, a 1 °C reduction in mean temperature below 11 °C was associated with an increase in mortality of 2.9%, 3.4%, 4.8% and 1.7% from all-causes, cardiovascular, respiratory, and non-cardio-respiratory causes respectively [9]. In Northern Ireland, a 1 °C fall in temperature during winter months resulted in a 4.5% increase in all-cause mortality among adults and a 3.9% and 11.2% increase in cardiovascular and respiratory deaths respectively [10]. In an analysis of deaths from natural causes in 15 European cities, the cold-related relative risk in London was higher than in the colder cities of Helsinki, Prague and Stockholm, but lower compared to most of the other cities studied [11]. However, the above results do not take into account the frequency of cold days experienced and so do not necessarily reflect total cold attributable burdens. An international comparison that quantified attributable fractions reported that cold was responsible for proportionally less deaths in the UK compared to China, Japan or Italy [12].

Low temperatures have also been associated with raised risks in morbidity outcomes in the UK, including emergency hospital admissions for respiratory diseases [13] and myocardial infarctions, [14] GP consultations for respiratory problems, [15] activation of implantable cardioverter defibrillators among cardiac patients, [16] and delayed ambulance call response times [17]. Most recently, the utility of using data from the Emergency Department Syndromic Surveillance System was demonstrated to provide timely indication of public health impacts in relation to cold weather [18].

##### Delayed effects

Cold effects can be delayed by up to a few weeks following initial exposure, originating the need to consider exposure effects over multiple days. In general, cold effects on cardiovascular diseases have been shown to be more immediate than on respiratory causes [19]. Unlike with hot weather, there is no evidence that there are additional impacts on health when cold conditions occur on consecutive days as part of a cold-spell [20]. Also unlike with heat-related deaths in the UK, there is little evidence of short-term displacement of cold-related deaths, indicating they do not predominantly occur in already frail individuals who may have been expected to die anyway within a short space of time [11].

##### Snow and ice

In general, there are increased rates of emergency hospital admissions for falls associated with harsh winter conditions [21]. Hip fractures among elderly groups are only weakly associated with snow and ice conditions in the UK, [22–25] with the great majority of falls resulting in a fractured hip occurring indoors. Fractures of the forearm and wrist are more strongly linked to cold and icy weather [26].

#### Vulnerable groups

A person’s susceptibility to cold weather is affected by both individual and contextual-level risk factors [27].

##### Age

The most important risk factor for cold-induced illness and death is advanced age [28]. An elevated mortality risk is observed in all age groups, but the risk increases steeply for elderly people. The elderly have reduced ability to thermoregulate their bodies and in other ways are also more susceptible to the effects of cold exposure [27]. Although snow and ice contribute to the risk of injuries and falls, the relationship is more complex when stratified by age. Younger, working-age adults are most sensitive to snow and ice-related fractures, [29] resulting in greater activity days lost. Risks associated with injuries tend to be comparatively low – an analysis of 21 emergency departments across England reported a 3.2% increase in adult trauma admissions associated with a 5 °C drop in minimum temperature, e.g. due to a severe night-time frost [30].

##### Pre-existing illness

Underlying diseases can modify blood pressure, circulation and perspiration rates, leading to increased cold risk [27]. Evidence for the UK generally indicates that respiratory diseases are the most sensitive to changes in temperature, but cardiovascular outcomes contribute a higher attributable burden because of the greater frequency of cardiovascular events [8, 31]. Specific cardiovascular diseases shown to be associated with cold include myocardial infarction [14] and stroke [32]. Among respiratory categories, chronic obstructive pulmonary disease (COPD) is strongly linked with wintertime weather and has been the outcome of intervention studies conducted within the home [33] and of preventative care informed by a health forecasting and alert system [34]. Many infectious diseases, including influenza, are seasonally distributed, but the contribution of weather factors to such outcomes is not clear.

##### Sex

Women may have slightly greater risk of cold-related death than men, but the difference is likely to be partly explained by differences in the age distribution. Women are also more likely to be living alone which can also heighten risk. An analysis of cold deaths in a cohort of elderly people from UK general practices found a greater risk in women: age-adjusted relative risk of 1.08 (95% CI 0.99 to 1.19) compared to men [28].

##### Rurality

The evidence for differences in effects by rurality is inconclusive. Excess winter mortality in the UK is reported not to vary by population density, [35] although living in a rural area may increase any effect of socio-economic deprivation [8].

##### Socio-economic status and fuel poverty

Evidence on the effect of socio-economic status is inconsistent, despite expected relationships between poverty, poor home heating and wintertime health [36]. No clear modification of risk by socio-economic gradient has been reported in several UK studies [8, 28, 37]. In the UK, social housing is often well heated and more energy efficient than housing in the owner-occupier and privately rented sectors [38]. However, the 2012 Hills review of fuel poverty and other reviews relevant to the UK refer to evidence on a range of health outcomes likely to be adversely affected by fuel poverty, including psychosocial wellbeing and quality-of-life measures [39–41].

##### Housing

Evidence on associations between housing and wintertime health is limited [42]. There is some observational evidence for the UK that vulnerability to cold is less in newer build and more energy efficient homes [38]. Analysis of data from the Health and Lifestyle Survey showed that, in Britain, housing quality tends to be worse in areas of colder climates, and that this leads to higher risk of hypertension [43]. In addition, a Cochrane Collaboration review on housing improvements concluded that interventions that raise thermal comfort in the home can lead to health improvements, especially when targeted at those with inadequate warmth and those with chronic respiratory diseases [44] This review also suggested that dwellings which are affordable to heat are linked to improved health and social relationships and may reduce absences from school or work. Furthermore, simulation studies of home energy efficiency improvement measures indicate net positive health benefits related to indoor temperature and indoor air quality, [45] although this may vary by setting [46].

#### Regional differences

Variations in the distributions of the above health, demographic, socio-economic and built-environment characteristics are likely to explain most differences in cold risk observed between UK regions. The most recent UK evidence on regional variations in cold risk are provided by an evaluation of the Public Health England Cold Weather Plan, [47, 48] as part of which epidemiologic assessment of retrospective data was conducted [29]. Adverse cold-related health impacts were observed in all regions, with the North East, North West and London experiencing the greatest relative increase in mortality. Impacts became apparent at fairly moderate values of mean temperature, indicating that ambient temperatures do not need to be particularly extreme before adverse effects occur. Evidence from Scotland suggests that other weather factors such as wind-chill have little additional effect on deaths once temperature has been modelled [9]. However, an apparent role for rainfall has been reported in explaining inter-town variations in mortality from ischaemic heart disease [49]. An assessment of GP consultations for respiratory conditions by elderly people in 16 locations across the UK observed the strongest risk in Norwich, although attributable fractions were highest in Edinburgh due to the Scottish city experiencing more cold days [50].

### Studies estimating current cold-related health impacts attributable to observed climate change (1970-2015)

Although cold-related health impacts reduced throughout much of the previous century in UK populations, there is little information on the role that milder winters due to climate change since the 1970s may have played in such reductions. As well as long-term reductions in seasonal mortality, [51–53] deaths in London specifically related to cold weather declined in a broadly uniform fashion throughout the twentieth century [54]. This study showed that the reduced vulnerability during the final period analysed (1986-1996) was consistent with the magnitude of reductions observed in the 3 earlier periods (all pre-1970s), suggesting that factors other than climate change may have been the main drivers. Furthermore, a global review of changes in population susceptibility to cold over time reported that there was little evidence for significant reductions in risk in the most recent decades [55]. An optimal detection analysis of deaths in England and Wales in those aged 50+ years reported that adaptation considerably enhanced previous reductions in cold-related deaths, with an estimated decrease of 85 cold-related deaths per million population per year, but only 47 if no adaptation had taken place [56].

One study from 1995 which used projections of temperatures from general circulation models to estimate future numbers of cold deaths for England and Wales reported that mean surface temperatures would increase 0.9 °C by 2010 (compared to the baseline period of 1968-1988) which would lead to 3310 less deaths during September-May, in the absence of any changes in health care or socio-economic conditions [57]. This estimate is lower than that of Alderson who predicted about 8000 deaths avoided for an increase of 1 °C in average winter temperature, although this number relates to excess winter deaths rather than those due specifically to cold [58].

As part of an assessment of the impact of climate change on winter road maintenance and traffic accidents in the West Midlands, it was reported that between 1999 and 2006 there was a general downward trend in the annual number of accidents, whilst over the same period there was no overall warming trend evident in mean winter temperatures [59]. This suggests that recent improvements in accident rates are due to non-climate related factors.

### Studies estimating future cold-related health impacts attributable to climate change during the periods 2015-2050 and 2050-2100

Estimates of current temperature-health risk profiles applied to future local climate data have been used in risk assessments to provide projections of future cold-related health impacts due to climate change, although as illustrated in the previous section there are likely to be other factors also that will contribute to eventual burdens. The studies below mainly consider an increase in mean winter temperatures compared to today. Although cold spells may also become more common in future due to increased climate variability, such sustained periods of low temperatures are not associated with additional mortality risk compared to individual cold days [20].

An assessment for the UK using an ensemble of 9 climate model realisations based on the medium emissions scenario (SRES A1B) reported that, in the absence of any adaptation by the population, a mean annual *increase* in cold-related deaths of 3% would be observed by the 2020s compared to current levels, followed by a decrease of 2% by the 2050s compared to current levels [60]. Cold risk remained highest in the elderly, in particular those aged over 85 years. An increase in deaths was observed for the 2020s due to projected demographic changes (increased population size and ageing) offsetting any reduction in impacts due to warmer winters. Had population size and age structure been held constant, then a reduction in cold-deaths of 9% and 26% was projected by the 2020s and 2050s respectively. Reductions are observed in all regions of the UK, with the highest cold-related death rate being in Wales (61.1 deaths per 100,000 people by the 2050s) and the lowest rate in Northern Ireland (34.1 per 100,000 people), although the latter rate was based on the current temperature-mortality risk profile of the neighbouring North West region due to data unavailability. The UK-wide cold-related death rate was 62.3 per 100,000 people for the 2020s and 50.6 for the 2050s.

A previous assessment for England and Wales reported an annual decrease in cold deaths of 6353 by the year 2030 and 8922 by 2050, assuming no changes in health care and socio-economic conditions [57]. About 40% of these avoided deaths were in deaths due to ischaemic heart disease, with the rest mainly from cerebrovascular disease, chronic bronchitis and pneumonia. Hames and Vardoulakis showed a decrease of 24% in cold-related mortality over the period 2020s-2050s, based on current population levels [61]. Other work, which also did not model future demographic changes, observed a 25% reduction in cold-related by deaths by the 2050s [62]. Separate estimates have also been produced for London as part of a global assessment [63].

The analysis of future burdens of traffic accidents in the West Midlands, using the UKCIP02 (UK Climate Impacts programme 2002) medium-high emission scenario, reported that a reduction in the number of days below 5 °C, leading to less days with slippery winter roads, will result in a reduction in the number of traffic accidents by 12% compared to a current annual baseline of 2039 accidents [59]. For later periods, the paper reports a 43% reduction in accidents by the 2080s, although continuing improvements in vehicle technology and road safety may mean that this is a conservative estimate.

Returning to mortality impacts, these were estimated to decrease by 26% over the period 2050s-2080s, assuming no population changes [61]. Hajat et al. reported a 40% decrease in the UK by the 2080s compared to current levels, but this decrease would only be 12% if future demographic changes are taken into account [60]. The UK-wide cold-related death rate for the 2080s was 40.9 per 100,000 people. An extension to this work reported that cold deaths in the UK by the 2080s will still outnumber heat deaths by a factor of over 4, assuming current susceptibility [64]. Although there are some arguments that climate change may not lead to a substantial reduction in winter mortality rates, [65, 66] the majority of assessments to date from the UK, which currently has a much higher burden from cold-related mortality than from heat-related mortality, project a greater reduction in cold-related deaths compared to the expected increase in heat-related deaths due to warmer summers. However, if temperatures continue to rise then heat-related impacts may eventually become more dominant [67]. There is little evidence from the UK on how climate change may affect rates of wintertime infections.

### Potential for impacts to be avoided by adaptation measures

Although milder winters due to climate change will likely mean that cold-related health impacts will reduce in future, there are likely to be other factors that will contribute to the changes. Despite population ageing and a progressive increase in the prevalence of cardiorespiratory disease, vulnerability to cold has reduced over the course of the previous century. [54] Although not quantified, the reasons for this likely include developments in health care (including influenza vaccination), [68] improved nutrition during winter months, better support services, and improved housing [51]. Indoor temperatures have risen as a result of improvements in building materials and standards, such that even today the age of a property remains an important determinant of indoor exposure, [69] which in turn broadly correlates with the risk of winter mortality [38] and morbidity [70]. However, these trends may be undone by future increases in fuel prices. In 2014, 2.38 million households, representing approximately 10.6% of all households in England, were in fuel poverty (defined as requiring fuel costs that are above the national median level) [71].

Factors that have reduced outdoor exposure to cold are also likely to have played a role in reducing vulnerability [52]. These include increased car ownership, climate-controlled transportation and shopping facilities, and improved clothing fabrics [72]. Measures such as windproofing bus shelters and better heating of waiting rooms could reduce the cold burden further [73]. It has been suggested that improved adaptation and acclimatisation to warmer summertime temperatures may increase vulnerability to cold in the future, although there is no evidence to support such maladaptation fears [55]. Although the number of traffic accidents related to wintertime weather are projected to reduce in future, the study authors warn that if a warmer climate results in budget cuts for highway maintenance then this may run the risk of reversing the declining trends [59].

Since the winter of 2011, England has had in operation a cold weather plan run by Public Health England (https://www.gov.uk/government/publications/cold-weather-plan-cwp-for-england). The plan provides advice for individuals, communities and agencies on how to prepare for and respond to severe cold weather, and initiates acute actions by health and social care professionals and other community organisations to strengthen the protection and support of vulnerable individuals when cold weather is forecast. As well as a recent process evaluation of this plan, [29, 47, 48] and possible future changes to the alert system employed, [74] further qualitative information has led to updates to the plan which the authors describe as an example of pragmatic evidence-based policy making [75]. To date, however, there is little quantitative evidence on the effectiveness of such plans either from England or elsewhere in the world. This lack of quantitative evidence also applies to most other types of cold-related health intervention measures. The arguable exception to this are home energy efficiency strategies, where there is enough evidence to suggest that these confer health benefits to some population groups [76–79].

## Conclusions

Although concerns about global warming have focussed recent attention on the dangers of exposure to hot weather, cold-related exposure remains a much greater public health problem in the UK. This may be exacerbated by future population ageing and possible increases in fuel prices. Although climate change is likely to lead to a reduction in future cold-related health impacts, intervention measures designed to minimise cold exposure and reduce fuel poverty will also play a key role in determining current and future health burdens associated with cold weather. The following evidence gaps are identified:

- There is no consensus on the role, if any, that deprivation plays on cold-related mortality and morbidity.
- With perhaps the exception of home energy efficiency interventions, there is limited quantitative evidence on the effectiveness of adaptation measures in reducing cold-related health impacts.
- The contribution that observed climate change since the 1970s has made on observed reductions in cold-related health burdens over previous decades needs to be better established.
- Although there is good information from risk assessments on the expected burdens of cold-related health due to climate change, future assessments could be improved by factoring-in spontaneous and planned adaptation options into estimates, and also by characterising the relative uncertainties contributed by the various elements of the assessment process.

### Open peer review

Peer review reports for this article are available in Additional file 1.

## Additional file

Additional file 1: Open peer review. (PDF 180 kb)
