# Correction to: Health effects of milder winters: a review of evidence from the United Kingdom

**DOI:** 10.1186/s12940-018-0353-6

**Published:** 2018-01-16

**Authors:** Shakoor Hajat

**Affiliations:** Department of Social & Environmental Health Research, London School of Hygiene & Tropical Medicine, 15-17 Tavistock Place, London, WC1H 9SH UK

## Correction

After publication of the article [1], it has been brought to our attention that there is an error in the abstract. The line that reads “a 1 °C fall during winter months led to reductions of 4.5%, 3.9% and 11.2%” should say “a 1 °C fall during winter months led to increases of 4.5%, 3.9% and 11.2%”.
