# Supplementary material for: Health effects of milder winters: a review of evidence from the United Kingdom
Source: Environ Health. 2017 Dec 5;16(Suppl 1):109. doi: 10.1186/s12940-017-0323-4 (PMC5773863; doi:10.1186/s12940-017-0323-4)
Supplement: Additional file 1: — Open peer review. (PDF 180 kb) [file 12940_2017_323_MOESM1_ESM.pdf]

Reviewer reports

Title: Health effects of milder winters: a review of evidence from the United Kingdom

Reviewer 1: Ho Kim

This paper reviews and summarizes health effects of milder winters in the United Kingdom. The author provides methodological problems of excess winter mortality indices and concludes that time-series regression or case-crossover are proper methods for unbiased inferences. He provides risks per 1°C fall of mean temperature below certain temperature (11°C for example). The reported risks are based on piecewise linear models, I assume. Because of the non-linear associations between temperature and mortality, adding some discussions of risk estimation would be helpful for general audience to have better understanding of this problem.

The author also provides useful information of vulnerability, current impacts, projected impacts, and adaptation measures. The conclusion are reasonable in general. But the overall impression of mine is that almost everything is uncertain now… I think it would be nice if the author could add something about what is almost certain and/or important to estimate future burden.

This manuscript provides useful up-to-date information in a well-structured manner about the health effects of milder winters in the UK. I think it is acceptable.

Declaration of competing interests:

I declare that I have no competing interests.

Reviewer 2: Yasushi Honda

To the author:

It is of great importance to assess cold impacts on health and how climate change affects the cold impacts. However, it is not appropriate to restrict the discussion to the UK only, because the journal is for international readers. After reading the manuscript, I think it can easily be modified such that "in the United Kingdom" can be deleted from the title. I suggest to do so, but if the author insists to keep the story within the UK, I think more explanations on how this can be generalizable to other areas.

I have one major comment and some minor ones as follows other than the above general comment:

Major comment

In the "Conclusions" section, the author correctly summarized the situation "There is little evidence on the contribution that observed climate change since the 1970s has made on observed reductions in cold-related health burdens over previous decades." If this is the summary, I think it is necessary to comment that the climate change impact projection based on the temperature-mortality relation has no basis after the descriptions on projection. Also, I do not think it is relevant (1) to state in the abstract that "Future climate change will likely result in a reduction in cold-related health burdens" without saying that the reduction is due to adaptation and (2) to speculate "climate change will lead to a net reduction in temperature-related deaths" in page 8, bottom lines

Minor comments:

1. Please spell out ONS in page 4, line 2.
2. In the "Mortality & morbidity outcomes" section, using relative risk is criticized and attributable fraction is introduced. I think the author can put this criticism at the end of this section; I feel a little odd to see explanations on relative risk again after the criticism.
3. In the "Age" subsection, it would be informative to readers to show the relative importance of injury & fall risk compared to, say, ischemic heart disease. The burden may not be very large for injury & fall, I suspect.
4. p.5, the last line "strongest association": I understand that this means higher relative risk, but strong association sometimes implies higher correlation coefficient or better fit, and using the word like relative risk would be more specific.

Declaration of competing interests:

I declare that I have no competing interests.

Author response

Dear Editor

Re: Health effects of milder winters in the UK

Thank-you for the useful reviewer comments on the above submission. The suggested changes have been incorporated in the revised manuscript in red text and are responded to below:

Reviewer 1: Ho Kim

This paper reviews and summarizes health effects of milder winters in the United Kingdom. The author provides methodological problems of excess winter mortality indices and concludes that time-series regression or case-crossover are proper methods for unbiased inferences. He provides risks per 1°C fall of mean temperature below certain temperature (11°C for example). The reported risks are based on piecewise linear models, I assume. Because of the non-linear associations between temperature and mortality, adding some discussions of risk estimation would be helpful for general audience to have better understanding of this problem.

Thank-you, this suggestion has been added to the 'Mortality and morbidity outcomes' section on page 4.

The author also provides useful information of vulnerability, current impacts, projected impacts, and adaptation measures. The conclusion are reasonable in general. But the overall impression of mine is that almost everything is uncertain now… I think it would be nice if the author could add something about what is almost certain and/or important to estimate future burden.

The evidence gaps have been slightly reworded to better convey that useful information is already available from risk assessments and how such assessments could be refined in future.

This manuscript provides useful up-to-date information in a well-structured manner about the health effects of milder winters in the UK. I think it is acceptable.

Reviewer 2: Yasushi Honda

To the author:
It is of great importance to assess cold impacts on health and how climate change affects the cold impacts. However, it is not appropriate to restrict the discussion to the UK only, because the journal is for international readers. After reading the manuscript, I think it can easily be modified such that "in the United Kingdom" can be deleted from the title. I suggest to do so, but if the author insists to keep the story within the UK, I think more explanations on how this can be generalizable to other areas.

The manuscript is based on an original review of evidence from UK populations. Opening-up the review to other settings would require new searches and additional text which would substantially delay and enlarge the manuscript. I decline this suggestion, however I do add a comment in the introduction section (page 3) to explain that the review is restricted to the literature on UK

populations but the evidence is likely to be generalisable to other high-income settings. Also the title of the paper has been slightly changed to clarify the scope.

I have one major comment and some minor ones as follows other than the above general comment:
Major comment
In the "Conclusions" section, the author correctly summarized the situation "There is little evidence on the contribution that observed climate change since the 1970s has made on observed reductions in cold-related health burdens over previous decades." If this is the summary, I think it is necessary to comment that the climate change impact projection based on the temperature-mortality relation has no basis after the descriptions on projection. Also, I do not think it is relevant (1) to state in the abstract that "Future climate change will likely result in a reduction in cold-related health burdens" without saying that the reduction is due to adaptation and (2) to speculate "climate change will lead to a net reduction in temperature-related deaths" in page 8, bottom lines

A comment has been added (page 8) after the current attribution section to acknowledge that future burdens are likely to be impacted by other factors in addition to climate change. The sentence from the abstract has been removed and the introduction sections. I agree that it is not helpful to talk about net impacts since both cold- and heat-related deaths will remain a public health challenge, and so this comment has been reworded (pages 8-9).

Minor comments:
5. Please spell out ONS in page 4, line 2.

This was defined in the abbreviations section but is now added to the main text (page 4).

6. In the "Mortality & morbidity outcomes" section, using relative risk is criticized and attributable fraction is introduced. I think the author can put this criticism at the end of this section; I feel a little odd to see explanations on relative risk again after the criticism.

This has been moved to the end of the paragraph (page 5). Thank-you for the suggestion.

7. In the "Age" subsection, it would be informative to readers to show the relative importance of injury & fall risk compared to, say, ischemic heart disease. The burden may not be very large for injury & fall, I suspect.

A reference has been added which quantifies this (page 5). I have also taken the opportunity to cite an additional paper which has been published since the last draft, and also to update the fuel poverty statistic to a more recent estimate and because the definition of fuel poverty has been changed by the Government.

8. p.5, the last line "strongest association": I understand that this means higher relative risk, but strong association sometimes implies higher correlation coefficient or better fit, and using the word like relative risk would be more specific.

This has been changed (page 6).
